# 725. Characteristics and Risk Factors for Mortality in Patients with Infections Caused by *Serratia marscescens* in a Referral Hospital in Nicaragua

**DOI:** 10.1093/ofid/ofad500.786

**Published:** 2023-11-27

**Authors:** Darwin Moisés Mayorga, Sunaya Marenco-Avilés, Guillermo D Porras-Cortés

**Affiliations:** Hospital Dr. Fernando Vélez Paiz, Managua, Managua, Nicaragua; Hospital Dr. Fernando Vélez Paiz, Managua, Managua, Nicaragua; Hospital Dr. Fernando Vélez Paiz, Managua, Managua, Nicaragua

## Abstract

**Background:**

*Serratia marcescens* is a relevant pathogen both for its ability to cause serious infections and for its resistance pattern. In Hospital Dr. Fernando Vélez Paiz (Nicaragua), *S. marcescens* is the second most frequent bacterium isolated in healthcare-associated infections. The aim of this study is to describe the epidemiology and characteristics associated with mortality in hospitalized patients with infections caused by this bacterium between January 2022 and December 2022.

**Methods:**

This is a retrospective, analytical, and cross-sectional study. The universe consisted of 239 patients with documented infections and positive cultures for *S. marcescens*. A sample of 131 patients was obtained once the inclusion and exclusion criteria were applied (Figure 1). An analysis of survivors and non-survivors was performed to identify risk factors for mortality.

**Figure 1.**
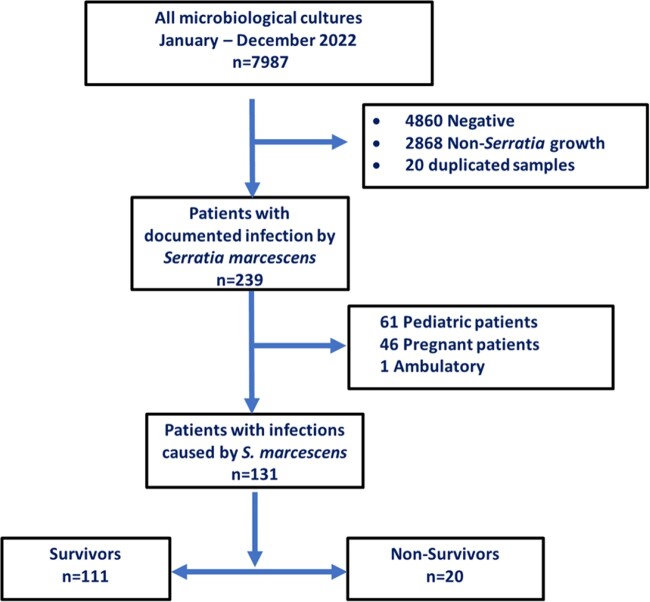
Flowchart of Selection of Patients with Serratia marcescens Infection

**Results:**

The mean age was 43.8 ± 17.1 years old, and 56.5% of the patients were female. At least one comorbidity was found in 53.4% of patients. The most common chronic conditions were diabetes (74.3%) and hypertension (58.5%). The most frequent sites of infection were surgical site (51.9%), skin and skin structure (24.4%), and bloodstream infection (10.7%) (Table 1). Of the 131 patients, 76 (58%) were categorized as having a healthcare-associated infection. The mortality rate was 15.3%. The non-survivors were older (56.5 ± 16.5 years old) than the survivors (41.6 ± 16.3 years old). The pneumonia was present in 15% of the non-survivors but only in 0.9% of the survivors. Extended-spectrum beta-lactamase (ESBL) production was detected in more than 80% of the strains of *S. marcescens*, and more than 50% were multidrug-resistant (Table 2). An analysis of multiple variables found factors associated with mortality such as: NEWS score ≥ 6 points [RM (95% CI): 84.0 (18.86-374.05)], cerebrovascular event and/or sequelae of this [RM (95% CI): 19.58 (1.92-199.3)], pneumonia [OR (95% CI): 19.41 (1.90-197.55)] among others (Table 3).

**Figure:**
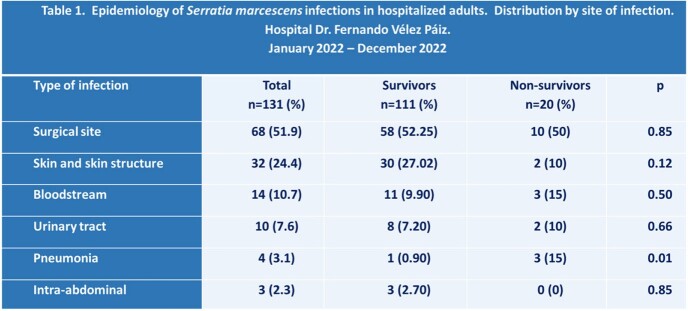

**Figure:**
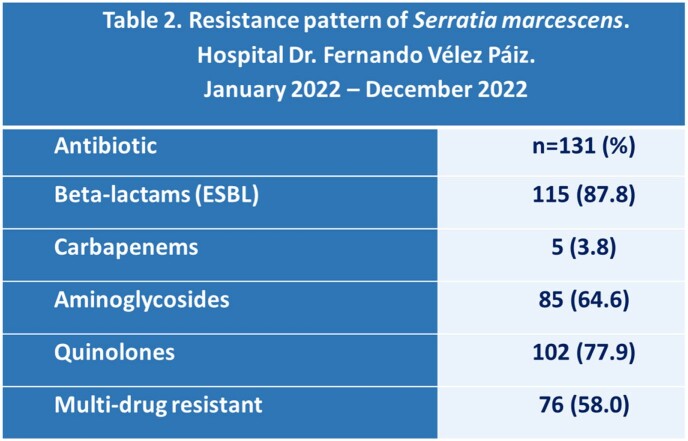

**Figure:**
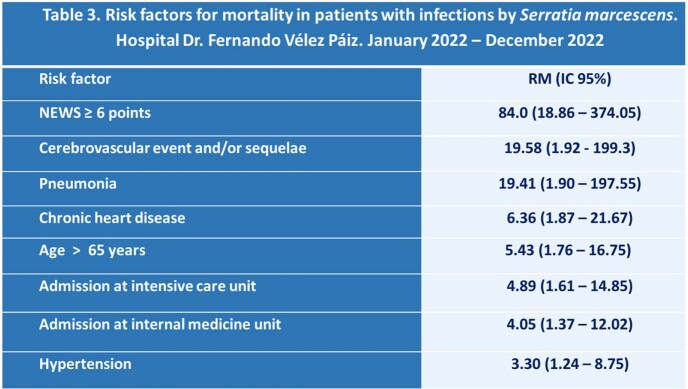

**Conclusion:**

*Serratia marcescens* infections were mainly surgical site, and of skin and skin structure. Pneumonia was associated with a higher chance of death. Most strains were ESBL producers. Different factors were associated with mortality, including severity on the NEWS score at the time of diagnosis of the infection.

**Disclosures:**

**All Authors**: No reported disclosures

